# Myopericytoma of the liver: A rare tumor - A case report

**DOI:** 10.1016/j.ijscr.2025.111110

**Published:** 2025-03-03

**Authors:** S. Kafle, S. Karki, A.D. Pant

**Affiliations:** Department of Pathology, Tribhuvan University Teaching Hospital (TUTH), Maharajgunj, Kathmandu, Bagmati Province, Nepal

**Keywords:** Myopericytoma, Liver tumor, Immunohistochemistry, Hemihepatectomy

## Abstract

**Introduction:**

Myopericytoma is an uncommon benign neoplasm composed of myoid-appearing perivascular cells. It had been reported in various soft tissues, but only few case reports were found reported in liver.

**Case presentation:**

We reported a case of myopericytoma in a 43-year-old female with a history of a left adnexal cyst and foul-smelling per vaginal discharge with no significant findings in per abdominal examination. Imaging studies initially suggested the possibility of hemangioma in ultrasonography and further CT scan was advised which gave the possibility of focal nodular hyperplasia.

**Clinical discussion:**

Right hemihepatectomy was performed and histopathological examination revealed a well-circumscribed mass consistent with myopericytoma, supported by positive immunohistochemical staining for SMA and H caldesmon, and negativity for Desmin, DOG-1, and ALK.

**Conclusion:**

This case contributes to the limited literature on hepatic myopericytoma and emphasizes the importance of considering this entity in the differential diagnosis of liver lesion.

## Highlights

• Myopericytoma is a rare benign neoplasm composed of myoid-appearing perivascular cells.
• Differentials in imaging range from hemangioma to benign tumor.
• Histopathological examination with immunohistochemical staining will help diagnose the disease and rule out malignancy.
• It had been reported in various soft tissues, but only few case reports were found reported in liver.
• Treatment is surgical excision of the lesion.

## 1 Introduction

Myopericytoma is a very rare benign perivascular neoplasm that arises from the perivascular myoid cells. Such neoplasm usually presents as slow growing, well-circumscribed, mostly painless firm nodule. Myopericytoma was first described by Granter et al*.* in 1998 as benign tumor with features of myoid line of differentiation [1]. However, the concept of a myopericyte was first proposed by Dictor in 1992 [2]. These neoplasms had been predominantly documented in soft tissues, with hepatic involvement being exceptionally uncommon. We reported a case presented in government university central hospital as a case of myopericytoma arising in the liver, detailing the clinical presentation, imaging findings, and histopathological features.

This case report has been reported in line with the SCARE criteria [3].

## 2 Case presentation

**Fig. 1:**
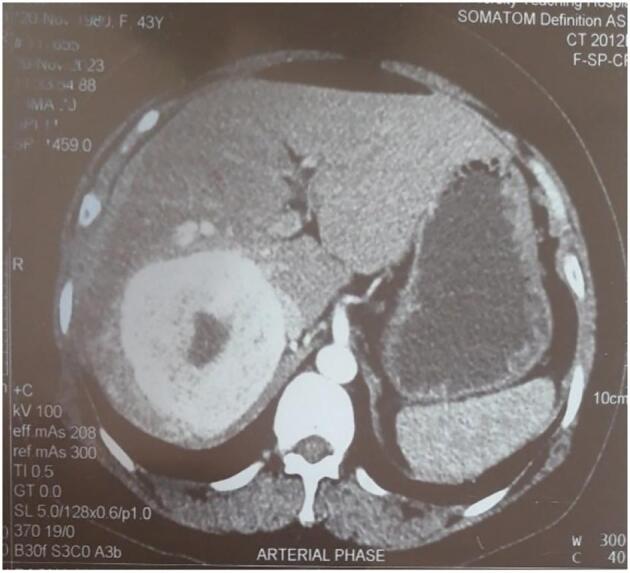
a: Imaging finding (CT scan) of the liver mass which showed a well-defined hypodense lesion in the right lobe of the liver, with a differential diagnosis of focal nodular hyperplasia.

A 43-year-old female presented in outpatient clinic with a history of a left adnexal cyst associated with foul-smelling PV discharge for the past 3–4 months. The patient was also under medication, tab. Thyroxine sodium-50 μg per day for hypothyroidism since 2 years. Physical examination revealed no abnormalities on per-abdominal assessment. The lesion in the liver was accidental finding during the ultrasonography of the abdomen and pelvis. Ultrasonography (USG) and CT scans identified a 9 × 8.7 × 8.4 cm well defined hypodense lesion in the right lobe of the liver, with a differential diagnosis of focal nodular hyperplasia in CT scan (Fig. 1). The patient got admitted and surgery (hemihepatectomy) was done.

### 2.1 Histopathological findings

In pathology department, a hemihepatectomy specimen was received. Gross examination of the specimen revealed a well-defined nodular growth measuring 9 × 7 × 5 cm within the liver, with a cut surface displaying a grey-white to brownish appearance. No areas of necrosis were observed.

**Fig. 2:**
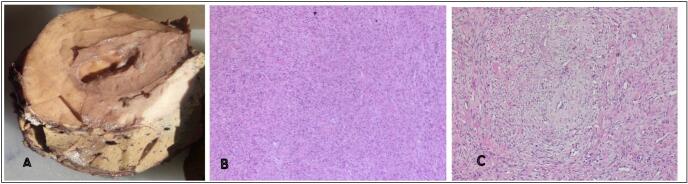
A: Gross image showing nodular mass in the liver. B: Microscopy finding at low power (50×) showing spindle-shaped tumor cells arranged in fascicles and numerous capillaries. C: Microscopy finding at high power (400×) showing spindle-shaped tumor cells arranged in fascicles and concentric pattern around numerous thin-walled capillaries.

Microscopic examination of multiple sections from the tumor mass revealed a well-circumscribed lesion characterized by spindle-shaped tumor cells arranged in fascicles and concentric pattern around numerous thin-walled capillaries (Fig. 2, A–C). The tumor cells were exhibiting elongated nuclei and moderate cytoplasm. No atypia, mitosis, or necrosis were observed. All submitted margins were free of tumor cells. Surrounding liver parenchyma did not reveal any significant morphological changes.

### 2.2 Immunohistochemistry

**Fig. 3:**
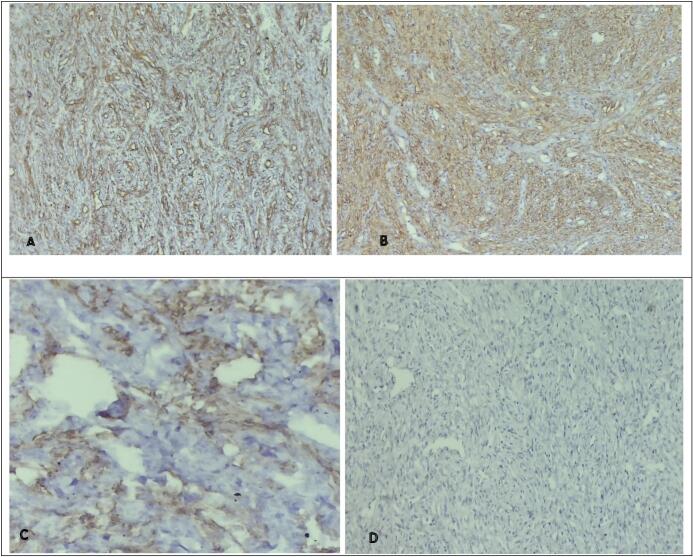
Immunohistochemical staining: A: CD34 highlighted blood vessel endothelial cells and were negative for tumor cells. B: tumor cells were positive for smooth muscle actin (SMA). C: tumor cells were patchy positive for h-caldesmon. D. Tumor was negative for desmin.

Immunohistochemical staining done were Smooth Muscle Actin (SMA), h-Caldesmon, Desmin, CD34, STAT6, DOG1, S100 and ALK. Our differentials include neural tumors like schwannoma, so S100 was negative, solitary fibrous tumors, CD 34 and STAT6 were negative and vascular tumor, CD 34 was negative. Positivity for SMA and h-caldesmon, confirmed the myopericytic nature of the tumor. CD34 highlighted blood vessel endothelial cells (Fig. 3). DOG-1, ALK and Desmin were negative, which ruled out the close differentials like GIST, Inflammatory myofibroblastic tumor and leiomyoma and further supporting the diagnosis of myopericytoma.

### 2.3 Diagnosis

Based on the clinical, radiological, and histopathological findings, the diagnosis of myopericytoma of the liver was established.

### 2.4 Therapeutic intervention

Based on the radiological findings, hemihepatectomy was already done. No further intervention was needed after histopathological report.

### 2.5 Follow-up and outcomes

As the lesion was benign and completely resected, the patient was completely recovered. There were no clinical signs and symptoms related to disease and ultrasonography of the abdomen and pelvis was normal on follow up at 6th months.

## 3 Discussion

Myopericytoma is a rare type of tumor with benign nature, and commonly affects all four limbs. The occurrence of myopericytoma in the liver is extremely rare [4]. In previous cases, duration of the lesion before excision were not known for most patients, and none of the lesions was painful [5]. Only in few cases, the clinical manifestation of a myopericytoma found involving the slow growth of painless nodules, and some patients with pain or tenderness [6]. In this case, the lesion was accidental findings during ultrasonography of the abdomen and pelvis for gynecological complaints. In the late stage, tumor size tends to increase significantly, and therefore, although most of the tumor are benign, early resection is still recommended [7]. The size of the tumor was 9 cm in dimension which was the first recorded case of such large size based on our knowledge. This might be due to delay in imaging due to absence of significant clinical sign and symptoms.

Imaging finding in USG showed a well-defined hypoechoic lesion with central hyperechoic area noted in segment V and VI of liver with internal vascularity. CECT of abdomen was suggested for definite opinion. CT scan of abdomen showed a well-defined round hypodense lesion noted in right lobe of liver and involving segment VI and VIII, and showed enhancement in arterial phase with non-enhancing component in the center, suggestive of scar. Also, showed progressive enhancement in subsequent porto-venous phase.

On histological examination, myopericytoma was considered by the presence of a distinctive concentric perivascular proliferation of round-to-spindle cells with a myoid appearance. As a tumor derived from perivascular myoid cells, myopericytoma was positive with muscle-specific actin and smooth muscle actin (SMA) [5]. In this case, histological features were quite clear with the concentric pattern (onion-skin like) of spindle shaped cells around numerous thin-walled capillaries. The capillaries were highlighted with immunohistochemistry (IHC) i.e. CD34 and the spindle cells by SMA. Further IHC (H caldesmon, Desmin, DOG-1, and ALK) were done for confirmation and to rule the close differentials like Angioleiomyoma, GIST and Inflammatory myofibroblastic tumor.

This case highlights the importance of considering myopericytoma in the differential diagnosis of liver nodules, especially when imaging studies suggest hemangioma or focal nodular hyperplasia. Another important point is to rule out malignancy with immunohistochemistry and imaging findings as myopericytoma are frequently misdiagnosed as other tumors, frequently sarcomas on histomorphology evaluation [5].

## 4 Conclusion

This case report emphasized the significance as a differential in case of hepatic lesion of benign nature. Histopathological findings, along with immunohistochemistry, plays a crucial role in confirming the diagnosis of hepatic myopericytoma.

## Consent

Written informed consent was obtained from the patient for publication and any accompanying images. A copy of the written consent is available for review by the Editor-in-Chief of this journal on request.

## Ethical approval

This study has been exempted from ethical approval by our institution.

## Guarantor

Shankar Kafle

## Research registration number

Not applicable.

## Funding

There is no any funding for this case report.

## Author contribution

Shankar Kafle: Visualization, Writing-Original draft preparation, data collection, data analysis or interpretation, writing the paper.

Shovana Karki: Validation, study design and review of the microscopic slides.

Anil Dev Pant: Editing and Supervision.

## Conflict of interest statement

There is no any conflict of interest.
